# A Single Protofilament Is Sufficient to Support Unidirectional Walking of Dynein and Kinesin

**DOI:** 10.1371/journal.pone.0042990

**Published:** 2012-08-10

**Authors:** Keitaro Shibata, Michi Miura, Yuta Watanabe, Kei Saito, Atsuko Nishimura, Ken'ya Furuta, Yoko Y. Toyoshima

**Affiliations:** Department of Life Sciences, Graduate School of Arts and Sciences, The University of Tokyo, Meguro-ku, Tokyo, Japan; Emory University, United States of America

## Abstract

Cytoplasmic dynein and kinesin are two-headed microtubule motor proteins that move in opposite directions on microtubules. It is known that kinesin steps by a ‘hand-over-hand’ mechanism, but it is unclear by which mechanism dynein steps. Because dynein has a completely different structure from that of kinesin and its head is massive, it is suspected that dynein uses multiple protofilaments of microtubules for walking. One way to test this is to ask whether dynein can step along a single protofilament. Here, we examined dynein and kinesin motility on zinc-induced tubulin sheets (zinc-sheets) which have only one protofilament available as a track for motor proteins. Single molecules of both dynein and kinesin moved at similar velocities on zinc-sheets compared to microtubules, clearly demonstrating that dynein and kinesin can walk on a single protofilament and multiple rows of parallel protofilaments are not essential for their motility. Considering the size and the motile properties of dynein, we suggest that dynein may step by an inchworm mechanism rather than a hand-over-hand mechanism.

## Introduction

Cytoplasmic dynein and kinesin move on microtubules (MTs) in opposite directions and play important roles in intracellular transport and organelle positioning [1]–[3]. Dysfunction of these proteins can lead to serious diseases and death [4], [5]. The molecular structure and functional mechanism of kinesin have been extensively investigated, and the hand-over-hand mechanism is generally accepted as the kinesin walking model [6], [7]. However, the dynein walking mechanism has not been elucidated despite several dissections of the mechanism [8], [9], although the structures are being solved [10]–[14]. Cytoplasmic dynein is a huge multisubunit complex (∼1.2 MDa) with a two-headed structure. The motor domain consists of a ring of six AAA modules and a coiled-coil stalk with a microtubule binding domain at the tip [15]–[17]. The dynein motor domain mass is ∼10 times larger than that of the kinesin motor domain, the diameter of the ring structure is ∼12 nm and the width is 10 nm [11], [12]. Dynein and kinesin step sizes have been measured as 8 nm [18]–[20] and their binding sites on the tubulin surface overlap around the helix 12 of β-tubulin [21]. A single MT consists of 13 protofilaments, and it has been assumed that walking dynein uses two or more protofilaments because of the large size of the dynein motor domain and the relative position between the two heads of dynein on a MT [8], [9]. Furthermore, dynein occasionally takes off-axis steps on MTs [20], [22], whereas kinesin walks along the MT longitudinal axis or follows the protofilament path with high fidelity [23], [24]. However, the minimum number of protofilaments required for supporting dynein and kinesin movement is unknown.

**Figure 1:**
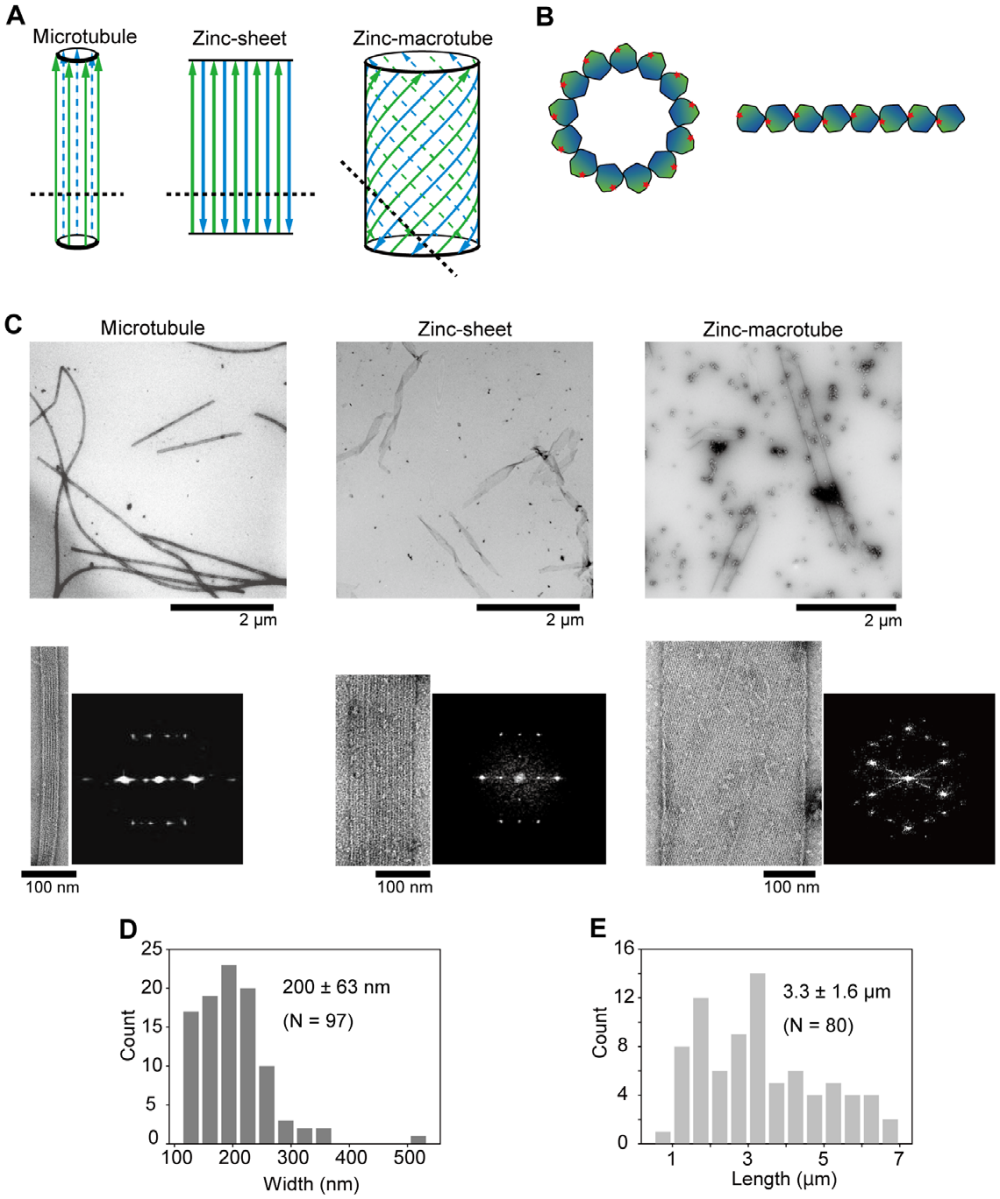
Structure of MTs, zinc-sheets and zinc-macrotubes. (**A**) The protofilament arrangements are modified from Wolf et al. (1993). Arrows represent the polarity of a protofilament, and color discriminates the outer side (green) from the inner side (blue) of a MT. Dashed lines indicate the cut plane of the cross-cut views in B. (**B**) The cross-cut views of a MT (left), zinc-sheet and zinc-macrotube (right). Red stars denote a helix 12 that is closely related to dynein and kinesin binding sites. (**C**) General views, close-up views and diffraction patterns of MTs (left), zinc-sheets (middle) and zinc-macrotubes (right). (**D** and **E**) The width and length distribution of zinc-sheets measured in EM images. The mean ± SD and number of counted zinc-sheets (N) are shown.

To elucidate the minimum number of protofilaments, we examined dynein and kinesin motility on zinc-induced tubulin polymers that consist of zinc-sheets and zinc-macrotubes. As schematically shown in Figure 1A, a zinc-sheet is arranged as adjacent protofilaments with anti-parallel and opposite-side orientations [25], [26]. Because lateral contacts between protofilaments in a zinc-sheet involve the helix 12 of tubulin [27], the helix 12 in dynein and kinesin binding sites should be unavailable. Therefore, binding sites are exposed on only one protofilament at one edge of a zinc-sheet (Figure 1B), and a zinc-sheet contributes a single protofilament as a track for these motor proteins. A zinc-macrotube is a wide cylindrical structure with a rolled zinc-sheet [28] and has no edge or binding sites (Fig. 1A). Thus, zinc-macrotubes are useful to confirm whether dynein and kinesin walk on protofilaments with unexposed helix 12 domains. Here, we report that a single protofilament is sufficient to support dynein and kinesin motility and discuss a dynein walking mechanism based on the results.

## Results

### Assembly of MTs, zinc-sheets and zinc macrotubes

Three types of tubulin polymers were assembled and examined by electron microscopy (EM) (Fig. 1C). Protofilament arrangements were also confirmed by their diffraction patterns (Fig. 1C). Based on EM images, the width of a zinc-sheet was 200±63 nm (± SD) (Fig. 1D) and the length was 3.3±1.6 µm (± SD) (Fig. 1E). The width of a MT was 30±2 nm (± SD, N = 20) and that of a zinc-macrotube was 293±18 nm (± SD, N = 20).

In a previous study, zinc-sheets under certain conditions often possessed a MT-like structure along one edge [28]. Therefore, we took utmost care so that polymerized zinc-sheets did not contain MT structures by reviewing the polymerizing conditions of Zn^2+^ and GTP concentrations, pH, incubation time and tubulin freshness. We mostly observed thin and folded ribbon-like structures, but did not find MTs or MT-like structures associated at the zinc-sheet edges in EM general views (Fig. 1C). As a control, authentically polymerized MTs were confirmed by EM (Fig. 1C).

However, there was a possibility that MTs polymerized in our zinc-sheet preparations and rarely resided on the carbon surface of an EM grid. To investigate this issue, we performed the following experiment. Cy5-labeled zinc-sheets and BODIPY FL-labeled MTs were polymerized individually, and then both polymers were mixed so that similar numbers of each were recognized by fluorescence microscopy (Fig. S1 A). The mixed solution was deposited onto a carbon grid for EM, and we observed a similar number of each polymer (Fig. S1B). Therefore, the possibility of hidden MTs was eliminated. Thus, we concluded that our zinc-sheet preparations did not contain MTs.

Another detail we addressed was zinc-sheet stability, because zinc-sheets tended to depolymerize in the motility assay solution with tubulin and Zn^2+^ concentrations lower than those under the polymerization condition. To prevent depolymerization, we stabilized zinc-sheets by fixation with glutaraldehyde (GA) for analysis in motility assays if necessary. The tubulin cross-linking ratio in the GA-treated zinc-sheets [zinc-sheets (GA)] was 46%, and GA-treated MTs [MTs (GA)] was 39% (Fig. S2). The formation of zinc-sheets (GA) was confirmed as identical to that of zinc-sheets in EM images.

### Gliding movements of MTs and zinc-sheets

**Figure 2:**
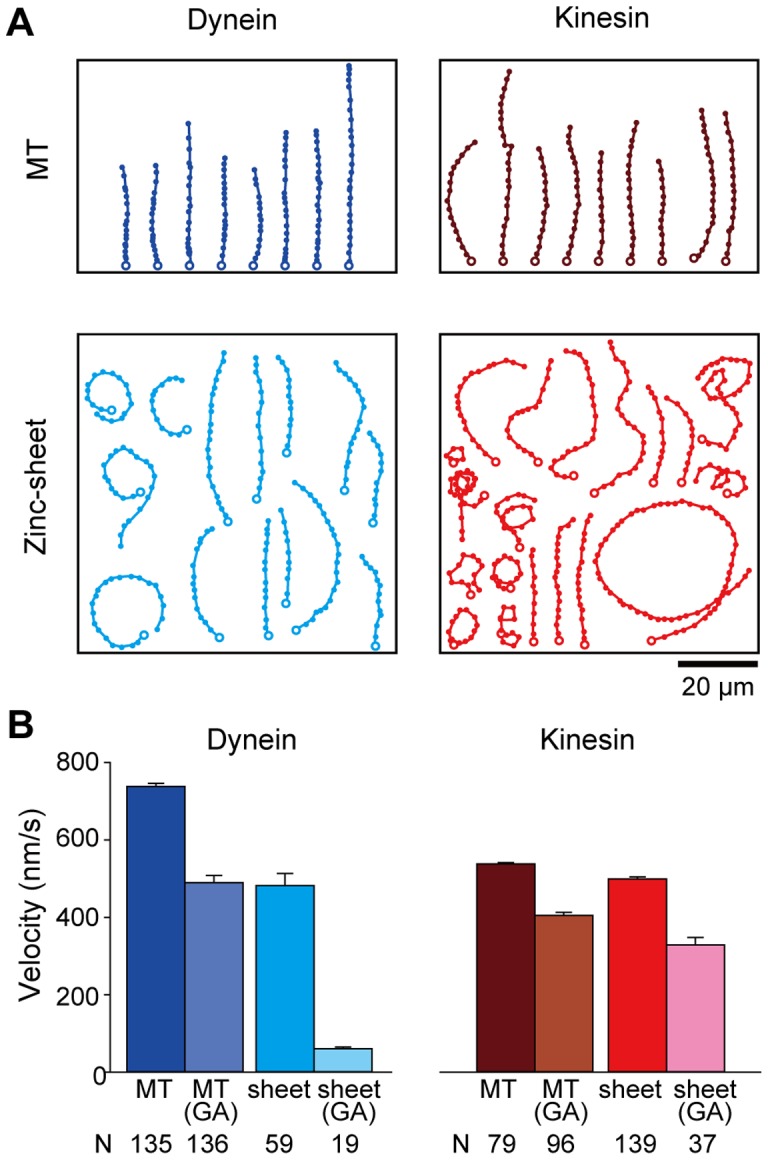
MT and zinc-sheet gliding movements on dynein- and kinesin-coated glass surfaces. (**A**) Movement trajectories of tubulin polymers plotted by positions marked every 3 s from the starting points (open circles). MT movements on dynein (upper left) and RK430-Avi (upper right). Zinc-sheet movements on dynein (lower left) and RK430-Avi (lower right). (**B**) Velocities of the gliding movement. Bars represent the mean ± SEM, and (N) is the number of measured gliding tubulin polymers.

In motility experiments, we examined whether tubulin polymers exhibited gliding on a glass surface coated with cytoplasmic dynein molecules purified from porcine brain or recombinant rat kinesin-1 dimers fused with an Avi-tag (RK430-Avi) (Fig. S2). Zinc-sheets as well as MTs moved on both surfaces (Fig. 2, and Videos S1, S2, S3, S4, S5, S6, S7, S8). Although MT gliding trajectories were mostly straight (Fig. 2A, upper), numerous zinc-sheet gliding trajectories were curved or circular (Fig. 2A, lower). Mean curvatures of the zinc-sheet trajectories on dynein and kinesin (Fig. S3C and G) were slightly larger compared with those of the MTs (Fig. S3A and E). The curved zinc-sheet trajectories were distinct from the straight MT trajectories, suggesting that the flexible ribbon-like structure of zinc-sheets can be driven by dynein and kinesin. Both GA-fixed polymers were confirmed to move on dynein- and kinesin-coated surfaces, but with decreased gliding velocities (Fig. 2B). Notably, the gliding fraction and velocity of a zinc-sheet (GA) on the dynein surface was greatly decreased, probably due to the folded and tangled structures of the zinc-sheet (GA) (Fig. 2B). In contrast, zinc-macrotubes bound to the dynein- and kinesin-coated surfaces, but did not show gliding movement. These results indicate that protofilament arrangements on the zinc-macrotube surface cannot support dynein and kinesin movement. Thus, the observed zinc-sheet gliding movement should be supported by a single protofilament at one edge of the sheet that exposes the motor domain binding sites.

To examine whether a zinc-sheet provides only one protofilament as a track for motor proteins, we observed zinc-sheets (GA) decorated with monomeric kinesin molecules [RK354 (G234A)] (Fig. S2), which bind to microtubules with high affinity, but is deficient in ATP hydrolysis and does not move on microtubules [29]. As shown in Figure S4A, kinesins appeared in a line at the far left protofilament. To confirm the kinesin repeat, diffraction patterns were obtained from the areas containing several protofilaments (Fig. S4B–G). Previously, MTs decorated with kinesins have shown a clear patterns of 8 nm longitudinal repeats [30]–[33]. Although the diffraction patterns of MTs do not show spots at the 8 nm layer line, those of zinc-sheets show weak spots at the 8 nm layer line (Fig. 1C) [25]. The areas containing the left edge of the kinesin-decorated zinc-sheet showed strong spots at the 8 nm layer line (Fig. S4B and C), and these 8 nm spots represented the kinesin repeat on the far left protofilament. The other areas without the left edge (Fig. S4D and E) or containing the right edge (Fig. S4F and G) showed obscure 8 nm spots, as did both edge areas of a non-decorated zinc-sheet (Fig. S4H and I). These results demonstrate that kinesin dominantly binds to one protofilament at one edge of a zinc-sheet, suggesting that a zinc-sheet contributes a single protofilament track for kinesin. Because dynein and kinesin binding sites on the tubulin surface overlap around the helix 12 of β-tubulin [21], a zinc-sheet also contributes a single protofilament as a track for dynein.

### Single molecule motility on MTs and zinc-sheets

**Figure 3:**
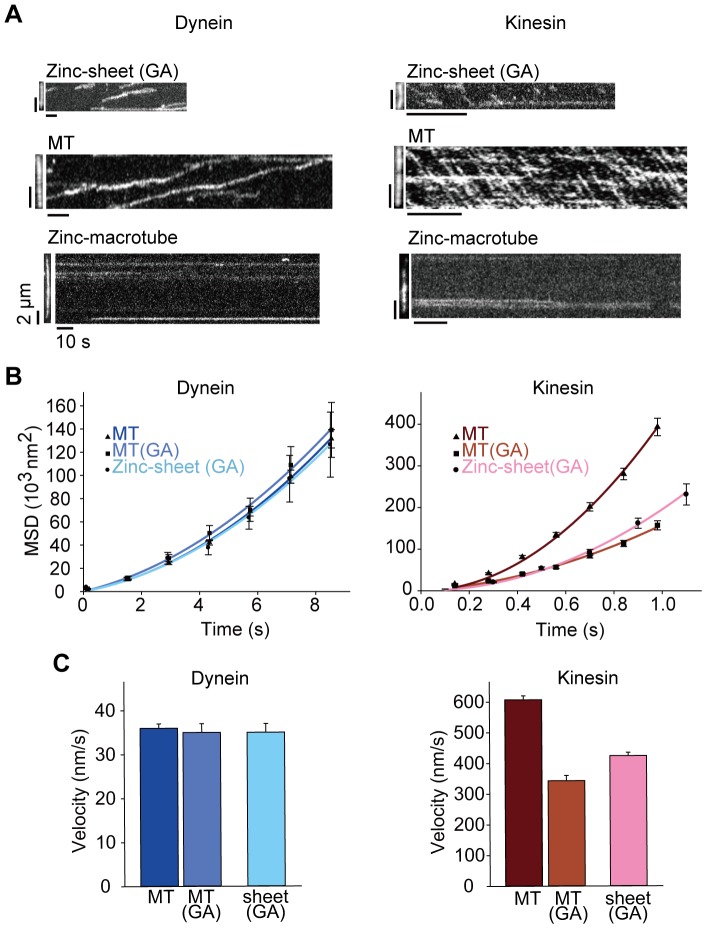
Single molecule motility of dynein and kinesin on tubulin polymers. (**A**) Tubulin polymers (left panels) and kymographs of GFP-GST-Dyn1_331kDa_ and RK430-GFP movements (right panels). (**B**) Mean-square displacement (MSD) plots of GFP-GST-Dyn1_331kDa_ (left) and RK430-GFP (right) movements on MTs, MTs (GA) and zinc-sheets (GA) with fitted quadratic curves (solid line). Each plot represents the mean ± SEM. (**C**) Velocities of GFP-GST-Dyn1_331kDa_ (left) and RK430-GFP (right) movements on MTs, MTs (GA) and zinc-sheets (GA) determined from MSD plots. Bars represent the mean ± SEM.

Next, a single molecule motility assay with dynein and kinesin was performed on the tubulin polymers. For this purpose, we used a recombinant yeast dynein dimer (GST-Dyn1_331kDa_) and a rat kinesin dimer (RK430) fused with GFP to visualize under a total internal reflection fluorescence (TIRF) microscope (Fig. S2). GFP-GST-Dyn1_331kDa_ is truncated at the tail domain, dimerizes via GST and walks on MTs processively at a similar velocity as that of full-length yeast dynein [20]. In gliding assay, porcine brain dynein was used, however, single molecules of porcine brain dynein do not walk processively and unidirectionally on MTs [34]–[36]. Therefore, this dynein cannot be used for a single molecule motility assay. Furthermore, because the zinc-sheets were liable to depolymerize during the TIRF microscopy observation, zinc-sheets (GA) were used. Under these conditions, single molecules of both dynein and kinesin moved processively and repeatedly on zinc-sheets (GA) (Fig. 3A, and Videos S9 and S10), and approximately half of the zinc-sheets (GA) in views contributed as tracks for the processive movement of dynein and kinesin. This track frequency (∼50%) was much higher than that of possible imperceptible MT contamination in our zinc-sheet preparations mentioned above. Therefore, dynein and kinesin were considered to move on zinc-sheets. Meanwhile, dynein and kinesin bound to one tip of a zinc-macrotube and were not observed to move (Fig. 3A). The result is consistent with that an exposed helix 12 protofilament is estimated to be approximately 70 nm at one tip of the zinc-macrotube, and that this length is not long enough to detect the processive dynein and kinesin movement in our assay system. Occasionally, dynein and kinesin also nonspecifically bound to some parts of zinc-macrotubes and did not move (Fig. 3A). Therefore, it appeared that the surface lattices of zinc-macrotubes and zinc-sheets did not support dynein and kinesin movement. From these observations, we concluded that dynein and kinesin walked on only one protofilament at the edge of zinc-sheets.

The velocities of the dynein movements on MTs, MTs (GA) and zinc-sheets (GA) were very similar [MT: 36±1 nm/s, MT (GA): 35±2 nm/s, zinc-sheet (GA): 35±2 nm/s] (Fig. 3C, and Table S1). These values were lower than that reported previously on MTs (102±32 nm/s) [20], because we used an assay buffer with a lower pH (pH 6.8) and lower salt concentration (25 mM potassium acetate) to improve the zinc sheet stability and the frequency of dynein movement. Additionally, it is known that the gliding velocity of MTs on yeast dyneins [20] is much slower than that on porcine brain dynein (Fig. 2B). Therefore, the dynein velocities obtained here are considered to be reasonable values, and the change of the assay conditions (lower pH and lower salt concentration) did not appear to significantly impair the dynein walking mechanism. It is note worthy that the dynein velocities on these three types of tracks were almost the same, suggesting that dynein motility is unaffected by GA fixation and the single protofilament available for movement.

We could not determine the statistical walking length of dynein and kinesin on zinc-sheets because EM showed infrequent tears and breaks in zinc-sheets, resulting in difficulty to assess whether dynein and kinesin were stochastically dissociated or structurally interrupted. However, the observed walking lengths were ∼500 nm (Table S1), indicating that both dynein and kinesin took more than 50 steps on a single protofilament.

Although the velocities of dynein were similar on MTs, MTs (GA) and zinc-sheets (GA), those of kinesin decreased with GA fixation (Fig. 3B and C). This difference may be attributed to dynein and kinesin binding sites on the tubulin surface, which overlap but are not identical. The velocity data suggest that kinesin is susceptible to GA fixation of the tubulin polymers. Moreover, it is possible that cross-linked sites in zinc-sheets after GA fixation are different from those in MTs, because their tubulin arrangements are different from each other. This difference in cross-linked sites might enable kinesin to move faster on zinc-sheets (GA) than on MTs (GA).

The velocities of dynein and kinesin on zinc-sheets are considered to be comparable to those on MTs. These properties of single molecule velocities were different from those of gliding velocities, which was probably due to the gliding movement being affected by the stiffness and distortion of the polymers and the number of motor proteins.

## Discussion

In this study, we achieved assembly of robust zinc-sheets without containing MTs. Next, we observed zinc-sheet gliding movements on dynein- and kinesin-coated glass surfaces and showed that zinc-sheets support dynein and kinesin motility. Finally, we observed dynein and kinesin motility on zinc-sheets and demonstrated that motor velocities on zinc-sheets are essentially identical compared with those on MTs.

Kinesin is known to walk along protofilaments of MTs [23], [24], however, whether a single protofilament by itself can support kinesin movement has not been elucidated. Kamimura and Mandelkow [37] demonstrated that zinc-sheets glide over a kinesin-coated surface. However, their zinc-sheets preparations were suspected to include MT-like structures [28], and they did not examine the processive movement of single molecules of kinesin on zinc-sheets. Our results obtained using a careful preparation of zinc-sheets and a single molecule assay directly show that the parallel arrangement of neighboring protofilaments is not required, indicating that kinesin can use only one protofilament of a MT for its processive movement.

On the other hand, dynein has been considered to use multiple protofilaments of a MT to walk, and occasionally takes off-axis steps over multiple protofilaments on MTs [20], [22]. Here, we demonstrated that a single protofilament is sufficient to support unidirectional and processive walking of dynein, in which single molecules of dynein move on a single protofilament processively.

**Figure 4:**
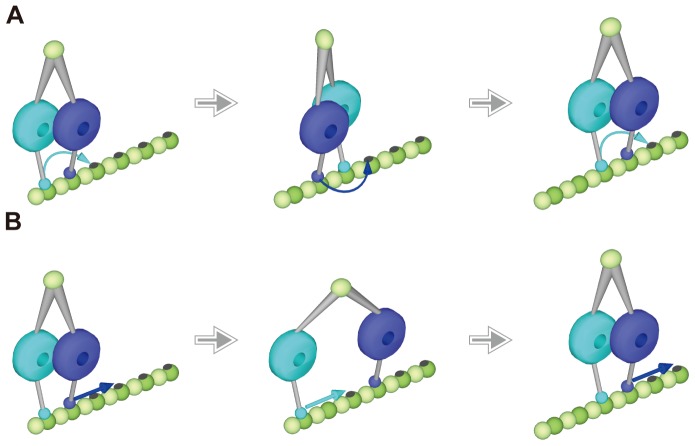
Schematic illustration of dynein walking on a single protofilament of a MT. (**A**) Partially overlapping hand-over-hand model. The rear head dissociates from the protofilament to overtake the front head and the two motor domains partially overlap side by side. (**B**) Inchworm model. The front head is always leading and the rear head is always trailing. Each head can slide and step advance without complete dissociation from the protofilament by keeping a weak interaction with the protofilament.

It should be noted that dynein velocities are almost identical on both a MT single protofilament of a zinc-sheet, because this finding suggests that dynein walks on a single protofilament without difficulty. Based on this notion, the dynein walking mechanism on a single protofilament is discussed. The diameter of the AAA ring of the dynein motor domain is ∼12 nm and the width is 10 nm [11], [12]. One head of a dynein molecule takes 16 nm steps, and the tail of dynein takes 8 nm steps [20]. Moreover, the plane of the ring motor domain is parallel to the MT axis [38]. To walk unidirectionally and processively on a protofilament, two dynein heads are considered to execute cooperative motion with each other. Specifically, as one head forms a weak association with a protofilament to take a step, the other maintains a tight association with the protofilament. If dynein walks by the hand-over-hand mechanism, as does kinesin, the two huge heads would physically hinder each other during alternate steps on the narrow track (Fig. 4A). On the other hand, the inchworm mechanism, where the front head is always leading and the rear head is always trailing (Fig. 4B), involves little physical hindrance. Because the two heads are capable of being apart at ∼40 nm [9], dynein can perform the inchworm motion without overlapping the two heads. Furthermore, in the inchworm mechanism, each MT binding domain can skate and step forward without dissociation from the protofilament, even in a weak binding state. Indeed, it has been previously reported that dynein shows this type of diffusional motion along the microtubule long axis in a weak binding state [34]–[36]. In the hand-over-hand mechanism, skating and stepping motions are difficult on a single protofilament, and each detachment and re-attachment to tubulin percross-bridge cycle does not favor processive movement. Therefore, we suggest that dynein should step on zinc-sheets by the inchworm mechanism rather than the hand-over-hand mechanism.

Recently, Dewitt et al. and Qiu et al. reported that the two heads of the dynein molecule are uncoordinated with each other while moving on MTs using multiple protofilaments, and the dynein heads do not pass each other in the majority of dynein steps [8], . In these studies, experiments were performed with dynein motor domains labeled with tags under a very low ATP concentration. However, some of those tags are similar in size to one AAA module, which may interfere with the head motion when one head passes over the other. Furthermore, the low concentration of ATP should increase the ATP waiting time during the cross-bridge cycle. These conditions may affect the stepping and coordination between heads. In our study, we used dynein molecules without extra decoration at motor domains under the physiological concentration of ATP, which eliminates the influence of such artifacts.

The stepping motion of dynein on the zinc-sheets discussed above might contribute to our understanding of the practical motion of dynein on MTs. Our results showing that the dynein velocities on both MTs and zinc-sheets are almost identical suggest the stepping mechanisms on both tracks are similar and dynein takes ‘inchworm-like’ steps on a MT by possibly using one or more protofilaments. Our findings do not exclude that dynein walks on a MT using multiple protofilaments by the hand-over-hand mechanism, but do show the highly flexible ability of dynein to processively walk on a single protofilament when the track is severely restricted. These findings might reveal one aspect of the versatility of dynein, which helps to perform its motor activity under various circumstances and plays many roles in cells.

## Materials and Methods

### Protein Preparation

Porcine brain as starting material to recover tubulin and dynein was purchased from Tokyo Shibaura Organ Co., Ltd. Other proteins were all recombinantly expressed and purified from *E. Coli* or *S. Cerevisiae*.

Purification methods are described in Materials and Methods S1.

### Assembly of MTs, zinc-sheets and zinc-macrotubes

MTs were polymerized from purified tubulin (3 mg/ml) as described previously [39]. Zinc-sheets were polymerized from 1 mg/ml tubulin in Zn^2+^ polymerization buffer (100 mM Pipes-KOH pH 6.1, 1 mM MgSO_4_, 0.5 mM EGTA, 4 mM GTP, 0.1 mM DTT, and 0.8 mM ZnSO_4_) by incubation for 1 h at 32°C. Zinc-sheets were stabilized with 20 µM paclitaxel. At pH 6.1, zinc-sheets are formed first and then roll up to form zinc-macrotubes after incubation for 10 h [26], [27]. Although zinc-sheets at pH 6.1 have a tendency to bend rather than remain straight, they were more resistant to depolymerization as compared with those at pH 5.5–5.9. Zinc-macrotubes were polymerized in the same manner as zinc-sheets, except the incubation was performed at 30°C for 20 h. Fluorescently labeled tubulin polymers were prepared by copolymerizing fluorescent tubulin and unlabelled tubulin at ratios of 1∶15–30.

The tubulin polymers were fixed with GA to maintain their stability for observation. Tubulin polymers were diluted with the polymerization buffer or Zn^2+^ polymerization buffer to 10%, and then the diluted solution was combined with 0.1% GA (G5882, Sigma). After incubation for 5 min at room temperature (RT), 100 mM Tris-Acetate (pH 7.5) was added to stop the cross-linking reaction. Fixed tubulin polymers were layered on a 20% sucrose cushion (10 mM Pipes-KOH pH 6.8, 50 mM potassium acetate, 4 mM MgSO_4_, 1 mM EGTA, 20 µM paclitaxel, 0.1 mM DTT, and 20% sucrose) and centrifuged at 14,000 ×g for 10 min at 27°C. The pellet was rinsed with 50 KAce assay buffer (10 mM Pipes-KOH pH 6.8, 50 mM potassium acetate, 4 mM MgSO_4_, 1 mM EGTA, 20 µM paclitaxel, and 0.1 mM DTT) and resuspended in the same buffer. Tubulin cross-linking ratios in tubulin polymers were determined by densitometry of SDS-PAGE using Quantity One (Bio-Rad).

### Electron Microscopy

Ten microliters of the tubulin polymers was applied to carbon-coated copper grids, stained with 1.5% uranyl acetate, and then observed under a transmission electron microscope (H-7500, HITACHI) with an accelerating voltage of 80 kV. Film images were scanned with a film scanner (LS-9000 ED, Nikon). The diffraction patterns were obtained by Image J.

In the zinc-sheet and kinesin binding experiment, polymerized zinc-sheets (GA) (10% final dilution) were mixed with RK354 (G234A) (0.13 µM final concentration) in 50 KAce assay buffer for 3 min.

### Gliding Assay of Tubulin Polymers

Gliding assays were performed in 50 KAce assay buffer supplemented with 1 mM ATP under a dark field microscope, as described previously [40]. Glass surface were coated with porcine brain cytoplasmic dynein and blocked by 5 mg/ml bovine serum albumin (BSA) (A0281, Sigma), or coated with biotinylated BSA (A6043, Sigma) to tether streptavidin (Wako) to bind RK430-Avi and then blocked with 7 mg/ml casein.

The ends of tubulin polymers were manually marked every 3 s using “Mark 2” software [41]. The curvatures of the gliding trajectories (µm^−1^, κ) were calculated according to κ = 1/*R* (where *R* represents a radius of curvature) in 5 µm (Fig. S3A–C and E–H) or 2 µm (Fig. S3D) fractions of each trajectory. The mean curvature was determined using nonlinear fitting of the cumulative probability distribution [41].

### Single Molecule Motility Assay on Tubulin Polymers

Fluorescently labeled tubulin polymers and GFP fusion motor proteins were observed by TIRF microscopy as described previously [41] at 23–25°C. The coverslips were silanized [42], and flow cells were constructed [41]. The flow cells were first coated with a 0.5% anti-β-tubulin monoclonal antibody solution (T5201, Sigma), allowed to absorb for 5 min, and then blocked with 1% Pluronic F-127 (P2443, Sigma) for 5 min, followed by 7 mg/ml casein (2242, Merck) for 5 min. Fluorescent tubulin polymers were then introduced and incubated for 5 min. After washing with 25 KAce assay buffer-(10 mM Pipes-KOH pH 6.8, 25 mM potassium acetate, 4 mM MgSO_4_, 1 mM EGTA, 20 µM paclitaxel, and 0.1 mM DTT), flow cells were filled with 25 KAce assay buffer containing 2–10 pM GFP fusion motor protein, 0.7 mg/ml casein, 0.1% Tween 20, 0.2% glucose, 1% 2-mercaptoethanol, 85 U/ml glucose oxidase (G2133, Sigma), 1,300 U/ml catalase (106810, Roche), and 1 mM ATP.

The positions of GFP fusion motor proteins were determined by a two-dimensional Gaussian fitting algorithm with “Mark 2” software [41]. For RK430-GFP, all runs were classified into moving and non-moving fractions by the variance per duration of interaction (VD; nm^2^/s) of 1,500 nm^2^/s for each run as described previously [43]. For GFP-GST-Dyn1_331kDa_, the VD was 700 nm^2^/sec calculated by tracking GFP-GST-Dyn1_331kDa_ absorbed non-specifically on the glass surface. Mean-square Displacement (MSD; ρ(τ)) was plotted by averaging the squared displacement for non-overlapping intervals τ. The velocity and the diffusion coefficient were determined by fitting ρ(τ) with ρ(τ)  = 2*D*τ + *v*2τ2 (where *D* represents the diffusion coefficient, and *v* is the drift velocity) [41].

### Microscopy image acquisition

A dark-field microscope (BX-51, Olympus) equipped with a 403/0.75 objective lens (UPlanFl, Olympus) was used for image acquisition. Images were magnified by a 53 TV adaptor, acquired with an intensified charge-coupled device (CCD) detector (ICD-6100, Ikegami), and recorded onto a hard disk via an analog/digital video converter (ADVC300, Canopus).

The TIRF microscope (IX71, Olympus) was equipped with a 1003/1.45 objective lens (PlanApo, Olympus). Images were magnified by IX2-SPT (Olympus), and captured by a back-illuminated CCD detector (DV887DCS-BV, Andor). GFP, BODIPY-FL were excited with a 488 nm argon ion laser (IMA101, Melles Griot) and Cy5 were excited with a 632.8 nm He-Ne laser (05LHP991, Melles Griot). The exposure time was 140 ms.

## Supporting Information

Figure S1 **Distribution of MTs and zinc-sheets in TIRF microscopy and EM images.** (**A**) Merged TIRF microscopy image of a mixture of fluorescently labeled MTs and zinc-sheets. MTs were polymerized with BODIPY FL-labeled tubulins and visualized as green, whereas zinc-sheets were polymerized with Cy5-labeled tubulins and visualized as red. Polymerized MTs were fragmented by sonication to be as short as zinc-sheets. (**B**) EM image of the same sample in (A). MTs and zinc-sheets were observed at an almost identical frequency compared with those in the TIRF microscopy image and, thus, it is unlikely that MTs had difficulty in binding to the EM grid while MTs were present in the mixed solution.
(TIF)

Figure S2 **The proteins used in this study.** (**A**) Schematic representation of the recombinant motor proteins. GFP was fused for detection by TIRF microscopy. An AviTag was fused for tethering kinesin to a glass surface. GST was fused to form a dimer of yeast dynein motor domains. His6 and FLAG were fused for protein purification. (**B**–**D**) SDS-PAGE images of the purified motor proteins. (**E** and **F**) SDS-PAGE images of cross-linked MTs and zinc-sheets by glutaraldehyde treatment. Multiple bands of cross-linked tubulin were weakly detected at the top of the gels (right parenthesis). Densitometric analysis of the gels revealed that the cross-linking ratios were 39% for MTs and 46% for zinc-sheets.
(TIF)

Figure S3 **Curvatures of tubulin polymer gliding trajectories.** (**A**–**H**) Histograms showing the curvature distribution of MT (**A** and **E**), MT (GA) (**B** and **F**), zinc-sheet (**C** and **G**) and zinc-sheet (GA) (**D** and **H**) gliding trajectories on a glass surface coated with multiple dynein (**A**–**D**) or kinesin (**E**–**H**) molecules. Curvatures were measured at 5 µm (except D) or 2 µm (**D**) fractions of each trajectory. Insets show the cumulative probability distribution. κ (µm^−1^) is the mean curvature determined by a cumulative probability distribution.
(TIF)

Figure S4 **Diffraction patterns of a zinc-sheet with kinesin.** (**A**) Negatively stained EM image of a zinc-sheet (GA) decorated with monomeric kinesin, RK354 (G234A) (Fig. S2), which binds to microtubules with high affinity, but is deficient in ATP hydrolysis and does not move on microtubules [1]. Several protofilaments at the edges of the zinc-sheet (GA) are numbered and the line of kinesin is marked as “K”. Scale bar  = 20 nm. (**B**–**G**) Diffraction patterns of the area shown by the left yellow box including the K and protofilament-1 (P1) (B), and parallel translations including a part of K, P1 and P2 (C), P1, P2 and P3 (D), and P2, P3 and P4 (E). Diffraction patterns of the area shown by the right yellow box including P2′, P3′ and P4′ (F), and parallel translation including P1′, P2′ and P3′ (G). (**H** and **I**) Diffraction patterns of both edge areas of a non-decorated zinc-sheet (GA) (micrographs, not shown). The bottom line represents equatorial axes, the middle line represents the 8 nm layer line, and the top line represents the 4 nm layer line. White arrows indicate 8 nm repeat spots.
(TIF)

Table S1 **Summary of single molecule motility.** The velocity and diffusion coefficient (mean ± SEM) were determined from MSD plots (Figure. 3B). The walking length on a zinc-sheet is the mean ± SEM of the walking length observed on a zinc-sheet (GA). N is the number of measured moving motor proteins.
(DOCX)

Video S1 **Gliding movement of MTs driven by dyneins.** Movement of MTs on a glass surface coated with porcine brain dynein molecules. Relatively straight trajectories are observed. Scale bar  = 2 µm. The movie speed is increased by 40-fold.
(MOV)

Video S2 **Gliding movement of MTs (GA) driven by dyneins.** Movement of MTs (GA) on a glass surface coated with porcine brain dynein molecules. Scale bar  = 2 µm. The movie speed is increased by 40-fold.
(MOV)

Video S3 **Gliding movement of zinc-sheets driven by dyneins.** Movement of zinc-sheets on a glass surface coated with porcine brain dynein molecules. A circular trajectory is observed. Scale bar  = 2 µm. The movie speed is increased by 40-fold.
(MOV)

Video S4 **Gliding movement of zinc-sheets (GA) driven by dyneins.** Movement of zinc-sheets (GA) on a glass surface coated with porcine brain dynein molecules. The movement is slow and irregular. Scale bar  = 2 µm. The movie speed is increased by 40-fold.
(MOV)

Video S5 **Gliding movement of MTs driven by kinesins.** Movement of MTs on a glass surface coated with recombinant rat kinesin molecules. Relatively straight trajectories are observed. Scale bar  = 2 µm. The movie speed is increased by 40-fold.
(MOV)

Video S6 **Gliding movement of MTs (GA) driven by kinesins.** Movement of MTs (GA) on a glass surface coated with recombinant rat kinesin molecules. Scale bar  = 2 µm. The movie speed is increased by 40-fold.
(MOV)

Video S7 **Gliding movement of zinc-sheets driven by kinesins.** Movement of zinc-sheets on a glass surface coated with recombinant rat kinesin molecules. Circular trajectories are mostly observed and others are relatively straight. Scale bar  = 2 µm. The movie speed is increased by 40-fold.
(MOV)

Video S8 **Gliding movement of zinc-sheets (GA) driven by kinesins.** Movement of zinc-sheets (GA) on a glass surface coated with recombinant rat kinesin molecules. Circular and figure eight trajectories are observed. Scale bar  = 2 µm. The movie speed is increased by 40-fold.
(MOV)

Video S9 **Movement of single dynein molecules on MTs, MTs (GA) and zinc-sheets (GA).** Movement of a single yeast dynein (GFP-GST-Dyn1331kDa) on Cy5-labeled MTs (left), MTs (GA) (middle) and zinc-sheets (GA) (right). The frames from a continuous GFP recording (green) were overlaid on the Cy5 image of the MT (red). Scale bar  = 2 µm. The movie speed is increased by 60-fold.
(AVI)

Video S10 **Movement of single kinesin molecules on MTs, MTs (GA) and zinc-sheets (GA).** Movement of single kinesin-GFP molecules on Cy5-labeled MTs (left), MTs (GA) (middle) and zinc-sheets (GA) (right). The frames from a continuous GFP recording (green) were overlaid on the Cy5 image of the MTs (red). Scale bar  = 2 µm. The movie speed is increased by 6-fold.
(AVI)

Materials and Methods S1 (DOCX)
