# Supplementary figures and images for: A Single Protofilament Is Sufficient to Support Unidirectional Walking of Dynein and Kinesin
Source: PLoS One. 2012 Aug 10;7(8):e42990. doi: 10.1371/journal.pone.0042990 (PMC3416812; doi:10.1371/journal.pone.0042990)

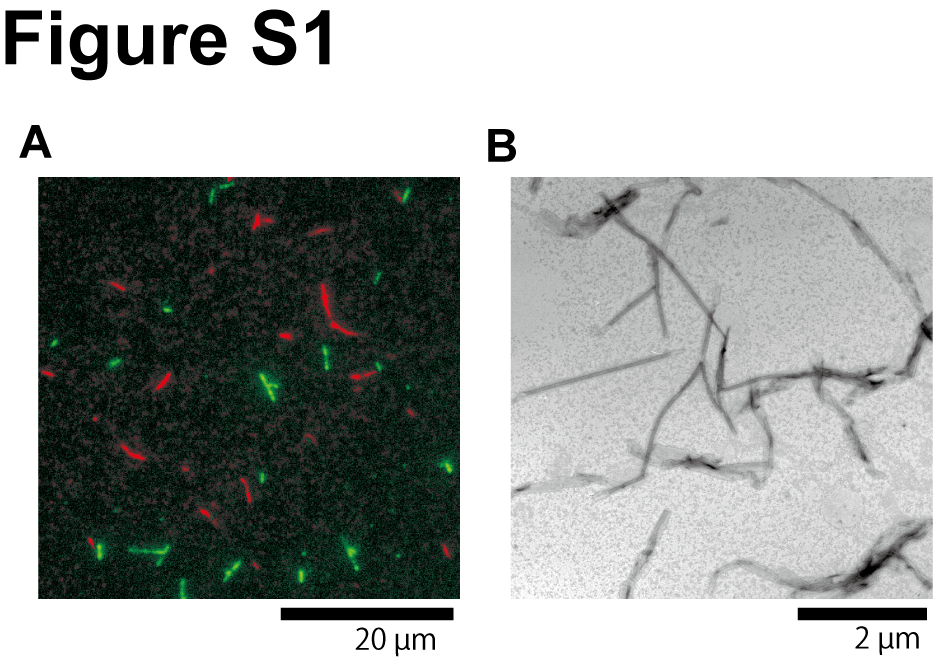

Supplement: Figure S1 — Distribution of MTs and zinc-sheets in TIRF microscopy and EM images. (A) Merged TIRF microscopy image of a mixture of fluorescently labeled MTs and zinc-sheets. MTs were polymerized with BODIPY FL-labeled tubulins and visualized as green, whereas zinc-sheets were polymerized with Cy5-labeled tubulins and visualized as red. Polymerized MTs were fragmented by sonication to be as short as zinc-sheets. (B) EM image of the same sample in (A). MTs and zinc-sheets were observed at an almost identical frequency compared with those in the TIRF microscopy image and, thus, it is unlikely that MTs had difficulty in binding to the EM grid while MTs were present in the mixed solution. (TIF) [file pone.0042990.s001.tif]

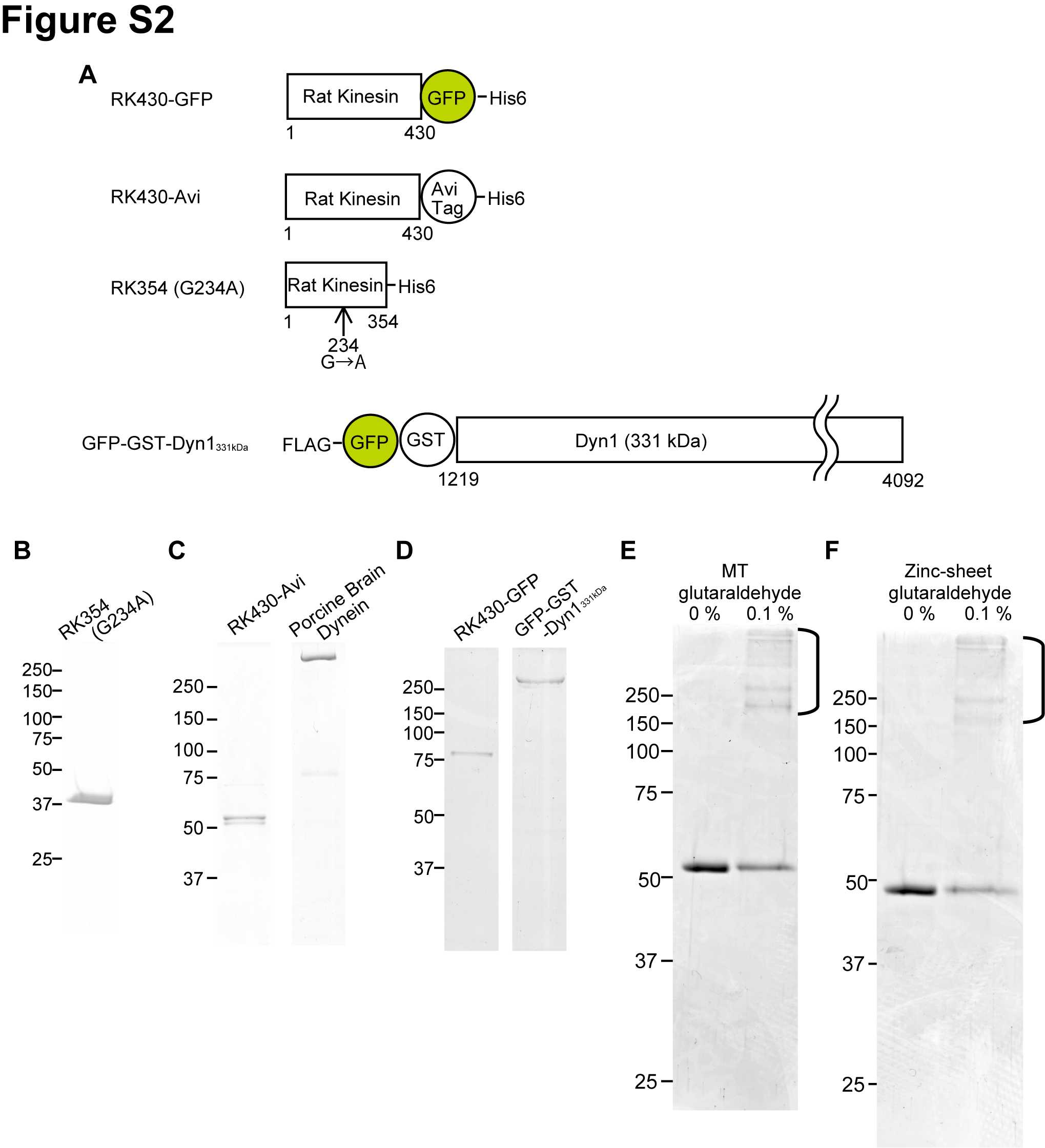

Supplement: Figure S2 — The proteins used in this study. (A) Schematic representation of the recombinant motor proteins. GFP was fused for detection by TIRF microscopy. An AviTag was fused for tethering kinesin to a glass surface. GST was fused to form a dimer of yeast dynein motor domains. His6 and FLAG were fused for protein purification. (B–D) SDS-PAGE images of the purified motor proteins. (E and F) SDS-PAGE images of cross-linked MTs and zinc-sheets by glutaraldehyde treatment. Multiple bands of cross-linked tubulin were weakly detected at the top of the gels (right parenthesis). Densitometric analysis of the gels revealed that the cross-linking ratios were 39% for MTs and 46% for zinc-sheets. (TIF) [file pone.0042990.s002.tif]

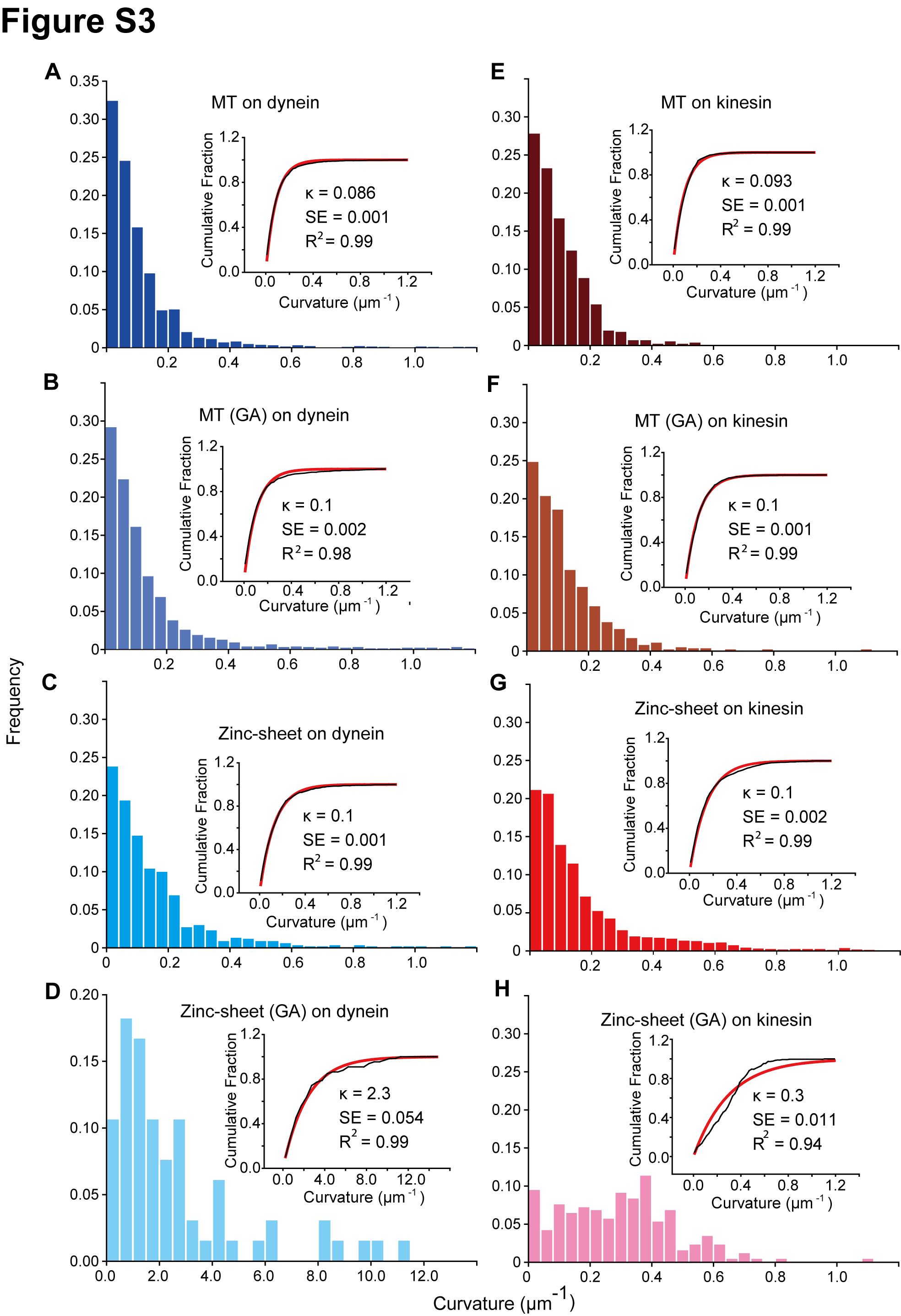

Supplement: Figure S3 — Curvatures of tubulin polymer gliding trajectories. (A–H) Histograms showing the curvature distribution of MT (A and E), MT (GA) (B and F), zinc-sheet (C and G) and zinc-sheet (GA) (D and H) gliding trajectories on a glass surface coated with multiple dynein (A–D) or kinesin (E–H) molecules. Curvatures were measured at 5 µm (except D) or 2 µm (D) fractions of each trajectory. Insets show the cumulative probability distribution. κ (µm−1) is the mean curvature determined by a cumulative probability distribution. (TIF) [file pone.0042990.s003.tif]

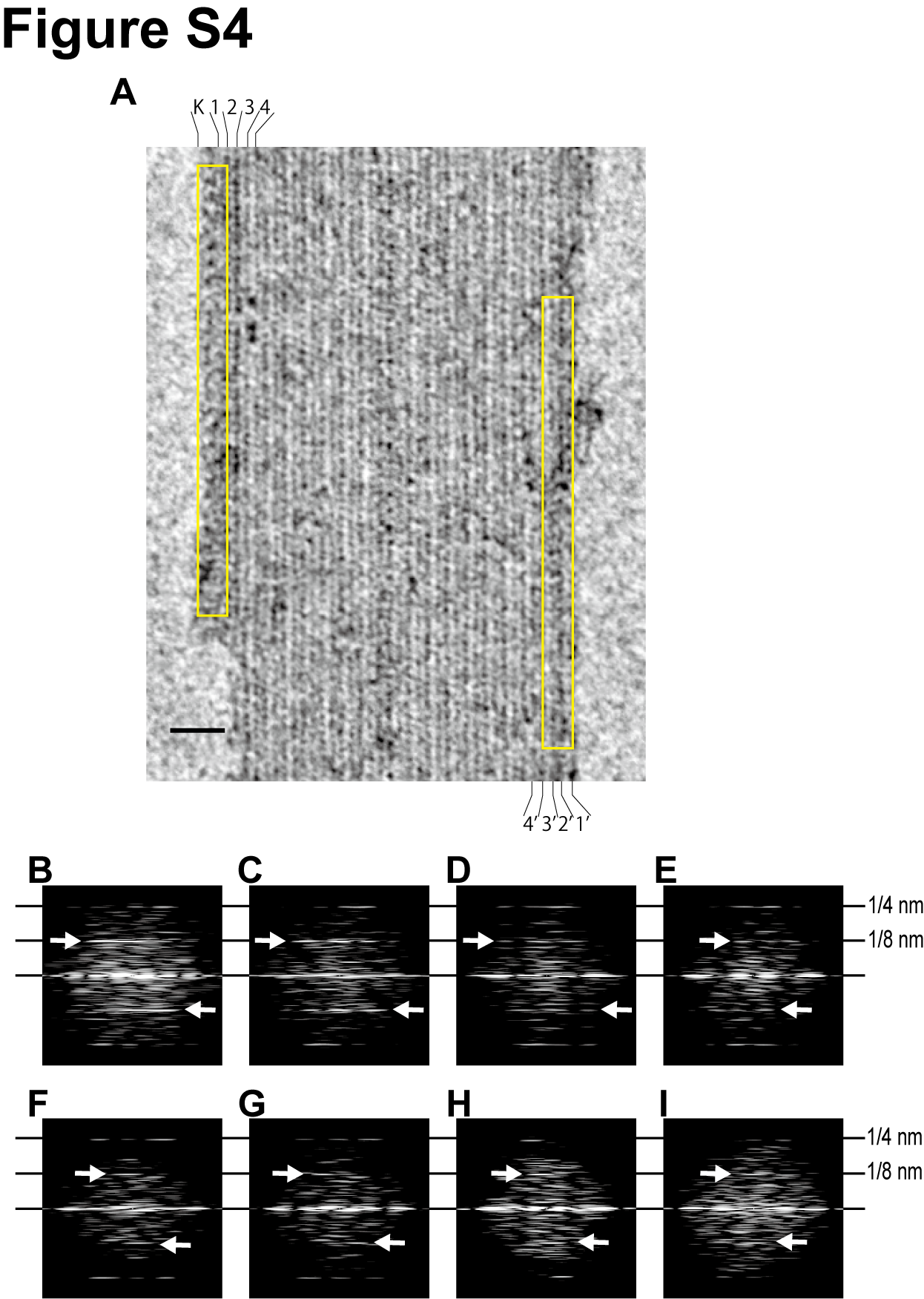

Supplement: Figure S4 — Diffraction patterns of a zinc-sheet with kinesin. (A) Negatively stained EM image of a zinc-sheet (GA) decorated with monomeric kinesin, RK354 (G234A) (Fig. S2), which binds to microtubules with high affinity, but is deficient in ATP hydrolysis and does not move on microtubules [1]. Several protofilaments at the edges of the zinc-sheet (GA) are numbered and the line of kinesin is marked as “K”. Scale bar = 20 nm. (B–G) Diffraction patterns of the area shown by the left yellow box including the K and protofilament-1 (P1) (B), and parallel translations including a part of K, P1 and P2 (C), P1, P2 and P3 (D), and P2, P3 and P4 (E). Diffraction patterns of the area shown by the right yellow box including P2′, P3′ and P4′ (F), and parallel translation including P1′, P2′ and P3′ (G). (H and I) Diffraction patterns of both edge areas of a non-decorated zinc-sheet (GA) (micrographs, not shown). The bottom line represents equatorial axes, the middle line represents the 8 nm layer line, and the top line represents the 4 nm layer line. White arrows indicate 8 nm repeat spots. (TIF) [file pone.0042990.s004.tif]
