# Supplementary material for: A Single Protofilament Is Sufficient to Support Unidirectional Walking of Dynein and Kinesin
Source: PLoS One. 2012 Aug 10;7(8):e42990. doi: 10.1371/journal.pone.0042990 (PMC3416812; doi:10.1371/journal.pone.0042990)
Supplement: Table S1 — Summary of single molecule motility. The velocity and diffusion coefficient (mean ± SEM) were determined from MSD plots (Figure. 3B). The walking length on a zinc-sheet is the mean ± SEM of the walking length observed on a zinc-sheet (GA). N is the number of measured moving motor proteins. (DOCX) [file pone.0042990.s005.docx]

**Table S1**

|  | | Velocity  (nm/s) | | Diffusion Coefficient  (x 10^4^ nm^2^/s) | Walking Length on zinc-sheet  (nm) | N |
| --- | --- | --- | --- | --- | --- | --- |
| Dynein-MT | 36 ± 1 | | 0.23 ± 0.02 | | − | 55 |
| Dynein-MT (GA) | 35 ± 2 | | 0.30 ± 0.04 | | − | 47 |
| Dynein-sheet (GA) | 35 ± 2 | | 0.23 ± 0.06 | | 429 ± 24 | 81 |
| Kinesin-MT | 607 ± 13 | | 1.71 ± 0.65 | | − | 52 |
| Kinesin-MT (GA) | 343 ± 17 | | 2.04 ± 0.48 | | − | 79 |
| Kinesin-sheet (GA) | 424 ± 11 | | 0.70 ± 0.43 | | 563 ± 24 | 86 |
