# Supplementary material for: A Single Protofilament Is Sufficient to Support Unidirectional Walking of Dynein and Kinesin
Source: PLoS One. 2012 Aug 10;7(8):e42990. doi: 10.1371/journal.pone.0042990 (PMC3416812; doi:10.1371/journal.pone.0042990)
Supplement: Materials and Methods S1 — (DOCX) [file pone.0042990.s016.docx]

**Materials and Methods S1**

**Preparation of Tubulin**

Tubulin was purified from porcine brain [2], and then stored in buffer (100 mM Pipes-NaOH pH 6.8, 2 mM MgSO_4_, 1 mM EGTA, and 0.6 mM GTP) in liquid nitrogen. To prepare fluorescently labeled tubulin polymers, tubulin was labeled with Cy5 (PA25001, GE Healthcare) or BODIPY-FL (D-6102, Invitrogen) [3]. Protein concentrations were determined by the Bradford method [4].

**Preparation of Brain Cytoplasmic Dynein**

In the gliding movement assay for dynein, we used mammalian cytoplasmic dynein prepared from porcine brain as described previously [5].

**Preparation of Yeast Cytoplasmic Dynein**

In the single molecule motility assay for dynein, we used a GFP fusion yeast dynein dimer that has the ability to move on MTs processively. Construction of the fused dynein was based on the truncated 331 kDa motor domain from *S. cerevisiae* dynein dimerized by GST [6]. To express and purify recombinant proteins, URA3, GAL, His6, FLAG, GFP and GST were added in frame with the start of the dynein 331 kDa motor domain coding sequence in the BY2777 strain (obtained from the Yeast Genetic Resource Center) [6].

Yeast cells were grown in SD medium to an OD_600_ of 1.4. The cells were harvested by centrifugation (9600 × *g*, 5 min, 4°C), and the cell pellet was resuspended in same volumes of dynein lysis buffer (50 mM HEPES-KOH pH 7.2, 200 mM potassium acetate, 2 mM magnesium acetate, 0.1 mM DTT, 0.5 mM ATP, 0.5 mM MgSO_4_, 0.05% triton X-100, and 10% glycerol) supplemented with cOmplete, Mini, EDTA-free (11 836 170 001, Roche). The solution was mixed with grass beads and vigorously vortexed. The resulting cell lysate was centrifuged at 300,000 × *g* for 15 min at 4°C. The resulting supernatant was then incubated with 40 µg/ml MTs and 10 µM paclitaxel for 10 min at RT. This mixture was layered on the 40% sucrose cushion in dynein lysis buffer supplemented with 10 µM paclitaxel and centrifuged at 300,000 × *g* for 20 min at 27°C. The resulting pellet was resuspended in dynein buffer B (50 mM HEPES-KOH pH 7.2, 200 mM potassium acetate, 2 mM magnesium acetate, 0.1 mM DTT, 0.05% Triton X-100, and 10% glycerol) supplemented with 400 mM KCl, 5 mM MgSO_4_, 3 mM ATP, 20 µM paclitaxel. After incubation for 10 min at RT, the mixture was centrifuged at 257,000 × *g* for 10 min at 27°C. The supernatant was then incubated with anti-FLAG IgG agarose (A2220, Sigma) for 1 h at 4°C. The IgG agarose was washed twice with dynein buffer B, and then bound dyneins were eluted with buffer B containing 0.5 mg/ml 3xFLAG peptide (F4799, Sigma). The eluted solution was aliquoted and frozen in liquid nitrogen.

**Preparation of Rat Kinesin-1 Fused with AviTag**

In gliding movement assay with kinesin, we used kinesin-1 dimer fused with AviTag (RK430-Avi), as described previously [7]. RK430-Avi was constructed by PCR from rat kinesin *rk430* clone (amino acid residues 1-430) and cloned into a modified pET-32a vector (Novagen) containing C-terminal AviTag (Avidity), linker amino acid sequence (KRCLE) and His_6_. The linker amino acid sequence between kinesin and AviTag is GSQLEFPG. The construct was expressed in *E. coli* BL21-CodonPlus (DE3) RIL (Stratagene), and RK430-Avi was purified as described previously [7], except that gel-filtration and MT affinity purification were not performed. The eluted RK430-Avi in 20 mM Na-Pi buffer (pH 7.5) containing 1 mM MgSO_4_, 300 mM NaCl, 0.1 mM ATP, 0.5 mM DTT and 300 mM imidazole was frozen and stored in liquid nitrogen.

**Preparation of Rat Kinesin-1 Fused with GFP**

In single molecule motility assay with kinesin, we used Rat Kinesin-1 dimer fused with GFP (RK430-GFP) which was known to exhibit processive motion. RK430-GFP was constructed by PCR from *rk430* clone and cloned into a pColdTF vector (Takara Bio) containing N-terminal His_6_, high soluble trigger factor (TF), HRV 3C protease site, Thrombin site, Factor Xa site. The linker amino acid sequence between kinesin and GFP is GSEF. RK 430-GFP was expressed and purified as described previously [7].

**Preparation of Monomeric Rat Kinesin-1**

Monomeric kinesin, RK354 (G234A), was constructed by PCR from the rat kinesin *rk354* clone (amino acids 1-354) and then cloned into a modified pET-32a vector (Novagen) containing a C-terminal His_6_. Then, 234 Gly was mutated to Ala. RK354 (G234A) was expressed and purified as described previously [7].

**Supporting References**

1. Rice S, Lin AW, Safer D, Hart CL, Naber N, et al. (1999) A structural change in the kinesin motor protein that drives motility. Nature 402: 778–784.

2. Weingarten MD, Lockwood AH, Hwo SY, Kirschner MW (1975) A protein factor essential for microtubule assembly. Proceedings of the National Academy of Sciences of the United States of America 72: 1858–1862.

3. Hyman A, Drechsel D, Kellogg D, Salser S, Sawin K, et al. (1991) Preparation of modified tubulins. Methods in Enzymology 196: 478–485.

4. Read SM, Northcote DH (1981) Minimization of variation in the response to different proteins of the Coomassie blue G dye-binding assay for protein. Analytical Biochemistry 116: 53–64.

5. Toba S, Toyoshima YY (2004) Dissociation of double-headed cytoplasmic dynein into single-headed species and its motile properties. Cell motility and the cytoskeleton 58: 281–289. doi:10.1002/cm.20018.

6. Reck-Peterson SL, Yildiz A, Carter AP, Gennerich A, Zhang N, et al. (2006) Single-molecule analysis of dynein processivity and stepping behavior. Cell 126: 335–348. doi:10.1016/j.cell.2006.05.046.

7. Furuta K, Edamatsu M, Maeda Y, Toyoshima YY (2008) Diffusion and directed movement: in vitro motile properties of fission yeast kinesin-14 Pkl1. The Journal of biological chemistry 283: 36465–36473. doi:10.1074/jbc.M803730200.
